# Family Coresidence, Parent–Child Interaction, and Deviant Behavior Among Rural Chinese Adolescents

**DOI:** 10.3390/bs16101767

**Published:** 2026-09-28

**Authors:** Lunxuan Sun, Wanli Mu

**Affiliations:** Faculty of Education, Tianjin Normal University, Tianjin 300387, China; genzhonghua@126.com

**Keywords:** family coresidence, parental noncoresidence, parent–child interaction, parental supervision, parent–child communication, adolescent deviant behavior, agricultural hukou, China Education Panel Survey

## Abstract

Family coresidence can structure adolescents’ access to parental guidance, but evidence often separates living arrangements from parenting processes. We analyzed 3612 rural-hukou adolescents from 110 schools in the China Education Panel Survey. Deviant behavior was measured as a five-item summed score (5–25). Primary ordinary least squares models used the untransformed score, school-clustered inference, and a core set of stable or plausibly pre-exposure covariates; expanded-control, log-outcome, multiple-imputation, item-level, and instrumental-variable models served as sensitivity analyses. Exploratory coefficient-product models assessed parental supervision and parent–child communication as parallel indirect associations. In the primary binary model, not living with both parents was associated with a 0.312-point higher score (95% CI [0.114, 0.510]). In the four-category model, the clearest multiplicity-adjusted association concerned adolescents living with neither parent (b = 0.388, 95% CI [0.148, 0.628]); estimates for the two one-parent groups were less precise. Multiple imputation supported the binary and neither-parent patterns. School-level instrumental-variable estimates were positive but imprecise and were interpreted as triangulation rather than causal identification. Lower supervision and less frequent communication formed statistically detectable indirect associations, although the cross-sectional design does not establish temporal mediation. Item-level analyses indicated that the composite association was not uniform across behaviors. The findings support a structural–process account while defining clear limits on causal and measurement interpretation.

## 1. Introduction

Across societies, adolescence brings greater autonomy without ending young people’s dependence on families for behavioral guidance. Parents help translate shared norms into everyday expectations, maintain knowledge of routines and relationships, and provide a relational setting in which conflict and difficult experiences can be interpreted. These functions are relevant to a broad range of externalizing and rule-violating behaviors because such conduct is shaped not only by individual disposition, but also by the continuity of behavioral boundaries and interpersonal access. Meta-analytic and longitudinal research connects parental behavioral control, monitoring, adolescent disclosure, and parent–child communication with externalizing problems, delinquency, and broader adolescent mental health (Kapetanovic et al., 2019; Ksinan & Vazsonyi, 2016; Pinquart, 2017; Zapf et al., 2024). This evidence raises a general question with relevance well beyond any single country: when parents and adolescents do not share everyday living space, which elements of parenting remain consequential for adolescents’ behavioral regulation?

Family coresidence provides a structural point of entry to this question: an adolescent may live with both parents, only the mother, only the father, or neither parent. Physical presence can facilitate observation, timely rule enforcement, and repeated conversation, yet coresidence is not equivalent to effective parenting. Nonresident parents may remain involved through telephone or digital contact and visits, whereas sharing a household does not ensure attentive supervision or open communication. Evidence on parental migration across low- and middle-income settings, as well as research distinguishing maternal, paternal, and dual-parent absence, demonstrates that parental separation varies in its configuration, duration, and associated outcomes rather than constituting a uniform family condition (Akezhuoli et al., 2022; Fellmeth et al., 2018; Lu et al., 2021). The analytically important distinction is therefore between family structure and family process: coresidence locates parents in adolescents’ everyday space, whereas supervision and communication indicate how parenting is enacted within or across that space.

### 1.1. Family Coresidence and Adolescent Deviant Behavior

The structural–process distinction is particularly salient for deviant behavior. In this study, the term refers to a broad domain of school-relevant interpersonal and institutional norm violations, including verbal aggression, fighting, bullying weaker classmates, truancy, and academic dishonesty. These behaviors differ in severity and immediate target, but each reflects difficulty complying with shared rules; the construct describes reported conduct rather than a fixed identity or clinical condition. Living apart from one or both parents may be associated with this behavioral domain through several mutually compatible processes. Reduced day-to-day contact can limit parents’ knowledge of school attendance, friendships, online activity, and return times; shifts to grandparental or other kin care can also require adolescents and caregivers to renegotiate rules and authority. At the same time, parental absence may coexist with economic remittances, continued emotional support, or compensatory caregiving. The expected association is therefore probabilistic rather than deterministic. A systematic review and meta-analysis linked parental migration to several adverse health outcomes among left-behind children and adolescents, including a small but statistically detectable increase in conduct-disorder symptoms; all included studies were observational (Fellmeth et al., 2018). More recent Chinese studies report higher deviant or health-risk behavior among adolescents experiencing non-intact family structure or parental absence (N. Wang et al., 2026; Y. Yang & Jiang, 2023).

A binary contrast between living with both parents and not living with both parents provides a clear overall test, but it can conceal substantively different arrangements. Mother-only and father-only coresidence retain one resident parent, whereas living with neither parent transfers most everyday care to other adults. Parent-specific migration studies find that the behavioral and mental-health correlates of maternal, paternal, and dual-parent absence do not always coincide (Akezhuoli et al., 2022), and sequence evidence suggests that prolonged dual-parent absence is among the family trajectories associated with less favorable development (Lu et al., 2021). Accordingly, we expect adolescents not living with both parents to report higher deviant behavior than those living with both parents (H1). We further expect this association to be most evident among adolescents living with neither parent, while treating the mother-only and father-only contrasts as distinct category-specific tests (H2).

### 1.2. Two Dimensions of Parent–Child Interaction: Parental Supervision and Parent–Child Communication

In this study, parent–child interaction refers to the recurrent exchanges through which parents and adolescents communicate behavioral expectations, information about everyday life, and responses to one another, thereby coordinating behavioral boundaries and mutual understanding. It is neither equivalent to the overall quality of the parent–child relationship nor inferable from coresidence alone. Family coresidence structures opportunities for everyday contact; parent–child interaction describes how parents and adolescents actually remain involved with one another within, or across, that spatial arrangement.

For the behavioral-socialization question examined here, two functions of this multidimensional process are analytically central. The first is behavioral regulation: parents establish expectations, manage domains of everyday conduct, and maintain behavioral boundaries. We capture this function through parental supervision. The second is information exchange: parents and adolescents discuss school experiences, interpersonal relationships, and personal concerns, allowing parents to remain informed and adolescents to receive explanation, advice, and responses. We capture this function through parent–child communication. The distinction is substantive rather than merely operational: supervision concerns how parents regulate conduct, whereas communication concerns how parents learn about and respond to adolescents’ experiences. The two dimensions also need not move together. Physical separation may restrict direct supervision without eliminating communication across distance, while coresidence may increase opportunities for contact without guaranteeing either process. We therefore measure supervision and communication separately, allow them to vary independently, and model them as parallel dimensions of parent–child interaction rather than combining them into a single composite score.

Parental supervision represents the behavioral-regulation dimension of parent–child interaction. It concerns how strictly parents regulate homework and examinations, school performance, friendships, clothing and appearance, time spent online, and television viewing. This form of behavioral control is conceptually different from psychological control, coercion, or intrusive manipulation. An updated meta-analysis associated appropriate behavioral control with fewer externalizing problems, whereas harsh and psychological control showed a less favorable pattern (Pinquart, 2017). Longitudinal evidence indicates that parental behavioral control is inversely associated with adolescent delinquency, that adolescent disclosure and delinquency are reciprocally related, and that discrepancies between youths’ and mothers’ monitoring reports predict later youth-reported, but not mother-reported, delinquency (Kapetanovic et al., 2019; Ksinan & Vazsonyi, 2016). Among rural Chinese adolescents, parental behavioral control was negatively correlated with bullying and victimization and predicted lower odds of membership in the combined bully–victim group, although the adjusted association did not extend to every bullying or victimization category (Chen et al., 2021). We therefore expect non-both-parent coresidence—especially living with neither parent—to be associated with lower parental supervision (H3a).

Parent–child communication represents the related but distinct informational dimension of parent–child interaction. Conversations about events at school, relationships with classmates and teachers, and private worries or troubles can help parents recognize emerging problems, clarify expectations, and provide advice before difficulties escalate. They can also invite adolescent disclosure, which is an important source of parental knowledge and a dynamic correlate of delinquency (Kapetanovic et al., 2019). Evidence from rural China indicates that parental migration is associated with greater emotional symptoms, conduct problems, hyperactivity, and total difficulties, whereas poorer parent–child communication is specifically associated with greater overall difficulties (F. Wang et al., 2019). Among Chinese children left behind by both migrant parents, Xie et al. (2024) identified five communication patterns combining phone or video contact with in-person visits. A systematic review further identifies adolescent-rated parent–child communication as a meaningful correlate of mental health across diverse populations, while noting that much of the evidence remains cross-sectional (Zapf et al., 2024). Because communication can occur across distance, it should be measured directly rather than inferred from coresidence. We expect non-both-parent coresidence, particularly living with neither parent, to be associated with less frequent parent–child communication (H3b).

Together, supervision and communication operationalize parent–child interaction without assuming that one process can stand in for the other. Supervision supplies behavioral regulation; communication supplies information exchange about school and personal experiences. A parent can regulate behavior without sustained discussion, and a nonresident parent can communicate frequently without directly observing everyday conduct. Examining the two processes in parallel therefore clarifies whether family coresidence is associated with deviant behavior through a regulatory pathway, a communicative pathway, or both. Consistent with evidence linking behavioral control and monitoring to externalizing outcomes, and parent–child communication to broader adolescent mental health (Kapetanovic et al., 2019; Pinquart, 2017; F. Wang et al., 2019; Y. Yang & Jiang, 2023; Zapf et al., 2024), we expect greater supervision and more frequent communication to be associated with lower deviant behavior and to statistically account for part of the coresidence–behavior association (H4).

### 1.3. Rural China as a Theory-Revealing Context

The value of rural China for this inquiry lies not only in the social importance of parental mobility, but also in its capacity to make a widely relevant structural–process problem observable at scale. Labor mobility, household-registration arrangements, extended-family caregiving, and communication across distance generate multiple coresidence configurations within a shared educational system. This setting therefore permits a stringent comparison between parents’ physical availability and the continuity of parenting practices. Although the institutional configuration is specific, the underlying question extends to transnational families and to employment-related, rotational, or other forms of nonresident parenting: does shared residence matter because it enables interaction, or can supervision and communication sustain behavioral regulation across distance? The broader literature on parental migration and communication shows why these alternatives must be separated empirically (Fellmeth et al., 2018; F. Wang et al., 2019; Xie et al., 2024).

Existing studies have established associations between family structure, parental absence, parenting processes, and adolescent outcomes (Akezhuoli et al., 2022; Huang & McKeown, 2022; Xu & Ren, 2025; Y. Yang & Jiang, 2023). Three issues remain central for a process-based account of school-relevant deviant behavior: whether an overall non-both-parent association is concentrated in a particular coresidence arrangement, whether supervision and communication form distinct parallel pathways, and whether the observed pattern is robust to alternative outcome, missing-data, covariate, and school-composition specifications. The present study addresses these issues using a large sample from the China Education Panel Survey (CEPS) of rural-hukou adolescents. It estimates binary and four-category measures of family coresidence, models parental supervision and parent–child communication in parallel, and uses multiple sensitivity analyses to evaluate the stability and boundaries of the observed associations.

## 2. Materials and Methods

### 2.1. Data Source and Study Sample

The China Education Panel Survey (CEPS) is a school-based survey designed and administered by the National Survey Research Center at Renmin University of China. Its original sampling design used stratification and multistage probability-proportional-to-size selection to draw 28 county-level units, 112 lower-secondary schools, 438 classrooms, and approximately 20,000 students. CEPS links questionnaires completed by students, parents or guardians, teachers, and school administrators. The present study constructed one cross-sectional analytic file by linking student, parent, household roster/background, school, and principal records through anonymized identifiers.

The student file contained 9449 original-cohort records, including 4843 adolescents with agricultural household registration. After cases with missing values on variables required for the common complete-case analysis were excluded, the analytic sample comprised 3612 adolescents nested in 110 schools across 28 county-level units. Of these, 2674 (74.03%) lived with both parents, 326 (9.03%) lived with their mother only, 121 (3.35%) lived with their father only, and 491 (13.59%) lived with neither parent.

Among the 4843 eligible adolescents, 61 lacked a complete five-item outcome and 1170 of the remaining 4782 had missing information on at least one other variable required for the common analysis. Complete-case retention was 76.77% for both-parent, 69.07% for mother-only, 62.69% for father-only, and 70.65% for neither-parent coresidence. Included and excluded adolescents were compared on observed characteristics, and multiple imputation was used as a sensitivity analysis (Supplementary Tables S1–S3, S6 and S9).

### 2.2. Measures

#### 2.2.1. Deviant Behavior

The outcome was based on the question, “During the past year, have you engaged in the following behaviors?” Five items assessed using profanity or verbally abusing others, fighting, bullying weaker classmates, skipping class or school, and copying homework or cheating on examinations. Responses were coded from 1 (never) to 5 (always) and summed when all five items were observed, producing a score from 5 to 25. The common sample showed modest internal consistency (Cronbach’s α = 0.663), consistent with a broad index spanning interpersonal aggression, rule violation, and academic dishonesty. The untransformed total was the primary outcome. Its natural logarithm was retained only as a sensitivity specification; no percentage interpretation was assigned to coefficients from the logged scale because the underlying score has no meaningful ratio zero.

#### 2.2.2. Family Coresidence

The binary measure was coded 0 when both parents lived at home with the adolescent and 1 when only one parent or neither parent lived at home. The expanded measure distinguished four mutually exclusive arrangements: living with both parents, living only with the mother, living only with the father, and living with neither parent. Living with both parents served as the reference category in all four-category models. The measure describes current household presence and does not assign a reason for noncoresidence.

#### 2.2.3. Parent–Child Interaction: Parental Supervision and Communication

Parent–child interaction was operationalized through two separately measured processes: parental supervision and parent–child communication. The scores were analyzed in parallel and were not combined into a single composite measure.

Parental supervision was measured with six questions asking how strictly parents regulated homework and examinations, school performance, friendships, clothing and appearance, time spent online, and time spent watching television. Each item was coded 1 (do not regulate), 2 (regulate, but not strictly), or 3 (regulate very strictly). Complete-item scores were summed to range from 6 to 18, with higher values indicating stricter supervision (Cronbach’s α = 0.736).

Parent–child communication was measured with eight questions covering whether the father and the mother separately discussed four domains with the adolescent: events at school, relationships with classmates, relationships with teachers, and private worries or troubles. Responses were coded 1 (never), 2 (occasionally), or 3 (often) and summed across the eight father- and mother-specific items, yielding a score from 8 to 24 (Cronbach’s α = 0.871). Higher scores indicate more frequent communication. A response concerning a nonresident parent was retained when reported because communication may occur across distance; unanswered items were treated as missing rather than recoded as an absence of communication. Both family-process scores were standardized within the common analytic sample before analysis.

#### 2.2.4. Covariates

Covariates were organized conceptually to limit overadjustment. Primary models included characteristics that were stable or plausibly preceded current coresidence: sex, age, only-child status, father’s education, mother’s education, and macro-region. Expanded-control sensitivity models additionally included the natural logarithm of one plus the number of close friends, academic achievement, psychological distress, parental relationship quality, father’s frequent drunkenness, an independent household toilet, perceived family economic condition, and school-area misconduct. These additional measures may improve observed covariate balance but may also partly reflect processes occurring after parental noncoresidence; accordingly, they were not treated as the sole basis for causal control. Coding details are reported in Table 1.

Table 1 reports descriptive statistics for the modeled outcome, family-coresidence measures, parent–child-interaction variables, and covariates in the common analytic sample. Every variable shown is complete for the same 3612 adolescents.

### 2.3. Statistical Analysis

#### 2.3.1. Baseline Models

Ordinary least squares models first estimated associations between family coresidence and the untransformed deviant-behavior score. The core-covariate specification was designated primary; the expanded-control model and the log-outcome model were sensitivity specifications. Binary and four-category exposures were estimated in parallel, with both-parent coresidence as the reference for category contrasts. Standard errors were clustered by school with finite-cluster correction. All tests were two-sided with α = 0.05. Within each four-category model, *p* values for the three nonreference contrasts were adjusted by Holm’s method. Results with 0.05 ≤ *p* < 0.10 were not considered statistically significant.

#### 2.3.2. Instrumental-Variable Strategy

As a sensitivity and triangulation analysis, two school-level instruments were constructed from the full linked student roster: the leave-one-out share of other students in the respondent’s school not living with both parents, and the corresponding share after the respondent’s classmates were excluded. Two-stage least squares models used the untransformed outcome, core covariates, and school-clustered finite-sample-adjusted standard errors; expanded-control results were examined separately. Relevance was evaluated with the first-stage coefficient, partial R^2^, and cluster-robust first-stage χ^2^. Because school-level parental noncoresidence may also reflect migration, socioeconomic context, peer composition, school resources, or behavioral climate, the exclusion restriction cannot be verified directly. The IV estimates are therefore interpreted as sensitivity evidence about direction and robustness, not as causal estimates.

For the four-category exposure, each nonreference arrangement was compared separately with the both-parent group using the corresponding leave-one-out school share and outside-class share. Holm adjustment was applied across the six category-specific second-stage tests. Because the father-only group was small and its first stages were weak, those estimates are reported for diagnostic transparency and are not interpreted substantively.

#### 2.3.3. Parallel Indirect-Association Analysis

The parent–child-interaction analysis used an exploratory parallel coefficient-product framework. Two a-path models regressed standardized parental supervision and standardized parent–child communication on family coresidence and core covariates. The total-association model regressed the untransformed deviant-behavior score on coresidence and core covariates, while the direct-association model simultaneously added both standardized family processes. Product terms (a × b) describe cross-sectional indirect associations; they are not interpreted as temporal mediatiors or mechanisms.

Uncertainty for indirect associations was estimated with 1999 school-cluster bootstrap replications. Percentile 95% confidence intervals and two-sided bootstrap *p* values are reported, with Holm adjustment across the 12 process-specific and combined indirect tests defined for this analysis.

#### 2.3.4. Missing Data, Sensitivity Analyses, and Software

Complete-case analyses used the same 3612 adolescents. Missingness was summarized for every modeled variable, complete-case retention was tabulated by coresidence category, and included and excluded cases were compared. A 20-imputation chained-equation sensitivity analysis was conducted among the 4782 adolescents with observed coresidence and outcome data; estimates were pooled using Rubin’s rules. Additional sensitivity analyses used the logged outcome and each of the five behavior items separately.

Analyses were conducted in Python 3.12.14 (Python Software Foundation, Beaverton, OR, USA) using pandas 3.0.5, SciPy 1.18.1, statsmodels 0.15.0, scikit-learn 1.9.1, and linearmodels 7.0. Primary tests concerned the raw-score binary and four-category core-covariate models. Expanded-control, log-outcome, multiple-imputation, and IV models were secondary sensitivity analyses; indirect and item-level analyses were exploratory. Holm adjustment was used within the related testing families specified above and in the Supplementary Materials.

## 3. Results

### 3.1. Family Coresidence and Deviant Behavior

As shown in Table 2, in the primary binary model, not living with both parents was associated with a 0.312-point higher raw deviant-behavior score (SE = 0.100, 95% CI [0.114, 0.510], *p* = 0.002). The estimate was attenuated in the expanded-control model (b = 0.155, SE = 0.081, 95% CI [−0.005, 0.315], *p* = 0.058). Sensitivity analyses using the logged score (b = 0.020, *p* = 0.034) and multiple imputation among 4782 adolescents with observed coresidence and outcome data (b = 0.243, 95% CI [0.100, 0.387], *p* < 0.001) preserved the positive direction.

In the primary four-category model, the multiplicity-adjusted association was clearest for neither-parent coresidence (b = 0.388, SE = 0.121, 95% CI [0.148, 0.628], Holm-adjusted *p* = 0.005). Mother-only coresidence was not associated with the outcome (b = 0.091, *p* = 0.399). The father-only estimate was positive (b = 0.597, unadjusted *p* = 0.035) but did not meet the multiplicity-adjusted threshold (Holm-adjusted *p* = 0.070). In the expanded-control model, the neither-parent coefficient remained positive (b = 0.219, unadjusted *p* = 0.035) but its Holm-adjusted *p* value was 0.105. Multiple imputation supported the neither-parent pattern (b = 0.359, Holm-adjusted *p* < 0.001).

### 3.2. Instrumental-Variable Analyses

Table 3 presents the binary IV sensitivity analyses using core covariates and the raw outcome. Both instruments strongly predicted not living with both parents (first-stage χ^2^ = 648.22 and 524.86; partial R^2^ = 0.118 and 0.113). The second-stage estimates were positive but imprecise: b = 0.644 (SE = 0.447, 95% CI [−0.233, 1.521], *p* = 0.150) for the same-school instrument and b = 0.691 (SE = 0.460, 95% CI [−0.211, 1.592], *p* = 0.133) for the outside-class instrument. Expanded-control estimates were similar in direction (Supplementary Table S8). The IV results therefore provide directional triangulation while retaining uncertainty about both precision and the exclusion restriction.

Table 4 reports category-specific IV diagnostics using the raw outcome and core covariates. Mother-only second-stage estimates were positive but imprecise. The father-only instruments were weak (first-stage χ^2^ = 5.13 and 1.34; partial R^2^ = 0.003 and 0.001), and their unstable second-stage estimates are not interpreted.

For neither-parent coresidence, both instruments had strong first stages and yielded similar positive second-stage estimates (b = 0.853, unadjusted *p* = 0.025; b = 0.852, unadjusted *p* = 0.022). After Holm adjustment across all six category-specific second-stage tests, both *p* values were 0.133. These estimates align in direction with the primary regression but are retained as sensitivity triangulation rather than separately identified causation.

### 3.3. Exploratory Indirect-Association Analysis: Parent–Child Interaction

The common sample of 3612 students was used for the exploratory pathway analysis. The analysis examined paths from family coresidence to standardized supervision and communication (a paths), associations of both processes with the raw behavior score in their joint outcome equation (b paths), and school-cluster bootstrapped indirect associations.

In the binary specification, not living with both parents was associated with lower parental supervision (a = −0.122 SD, SE = 0.042, *p* = 0.005) and less frequent parent–child communication (a = −0.246 SD, SE = 0.045, *p* < 0.001). In the joint outcome equation, one-SD increases in supervision (b = −0.381, SE = 0.055, *p* < 0.001) and communication (b = −0.290, SE = 0.051, *p* < 0.001) were associated with lower raw behavior scores.

The four-category a paths showed lower communication in all three nonreference groups and lower supervision most clearly in the father-only and neither-parent groups. Because the father-only group contained only 121 students, its path estimates are reported but interpreted cautiously. Table 5 presents these exploratory pathway estimates.

For the binary specification, the total association was 0.312 (SE = 0.100, *p* = 0.002) and the direct association after both interaction measures entered was 0.194 (SE = 0.093, *p* = 0.038). The combined indirect association was 0.118 (95% bootstrap CI [0.066, 0.172], Holm-adjusted *p* = 0.012), comprising supervision (ab = 0.046, 95% CI [0.013, 0.079], adjusted *p* = 0.020) and communication (ab = 0.071, 95% CI [0.038, 0.110], adjusted *p* = 0.012).

In the four-category specification, the neither-parent total association was 0.388 (SE = 0.121), and the direct association was 0.264 (SE = 0.115) after both interaction measures were added. The corresponding coefficient change was 0.124.

For the neither-parent group, the combined indirect association was 0.124 (95% CI [0.065, 0.194], Holm-adjusted *p* = 0.012). The supervision component was 0.051 (95% CI [0.012, 0.094], adjusted *p* = 0.040), and the communication component was 0.073 (95% CI [0.036, 0.121], adjusted *p* = 0.012). These products describe cross-sectional statistical accounting and do not establish temporal mediation. Table 6 reports total (c) and direct (c′) associations.

## 4. Discussion

This study examined associations among family coresidence, parent–child interaction, and deviant behavior in rural Chinese adolescents. Four findings stand out. First, not living with both parents was associated with a higher raw behavior score in the primary core-covariate model, although the estimate was attenuated in the expanded-control specification. Second, the clearest multiplicity-adjusted category pattern concerned adolescents living with neither parent and was supported by multiple imputation. Third, school-level IV estimates were directionally positive but statistically imprecise and depend on an untestable exclusion restriction. Fourth, lower supervision and less frequent communication formed parallel cross-sectional indirect associations. Together, the results support a bounded structural–process interpretation rather than a definitive causal or temporal mechanism claim.

### 4.1. Family Coresidence as a Configuration of Parental Availability

The binary result is consistent with prior evidence linking parental absence, migration, and non-intact family arrangements to conduct problems and other behavioral risks (Fellmeth et al., 2018; Ma et al., 2024; N. Wang et al., 2026; Y. Yang & Jiang, 2023). Its substantive meaning, however, becomes clearer only when the binary exposure is decomposed. The adjusted association was most evident for adolescents living with neither parent; mother-only and father-only coresidence did not show comparably precise adjusted differences from both-parent coresidence. This distribution is compatible with research showing that parental migration is heterogeneous and that paternal, maternal, and dual-parent absence may carry different correlates (Akezhuoli et al., 2022; Lu et al., 2021; Ma et al., 2024). It also extends research on left-behind adolescents by showing that the average non-both-parent contrast can mask where the association is concentrated.

This concentration is theoretically important because the distinction between one and no resident parent is not merely numerical. When one parent remains in the household, an adolescent may retain a direct line of everyday authority, routine coordination, and rapid response to school or peer-related events. When neither parent is resident, these functions must be divided among nonresident parents and other caregivers, often across households and communication channels. Such an arrangement can still be caring and effective, but it places greater coordination demands on the family. Recent studies have similarly found associations between dual-parent migration and aggression or other adjustment difficulties, although the size and form of the association vary by outcome and design (Ma et al., 2024; F. Yang et al., 2022). The present findings therefore should not be read as assigning a fixed deficit to a family type. Rather, they identify neither-parent coresidence as the arrangement in which maintaining continuous parental availability may require the most deliberate organizational effort.

The mother-only and father-only estimates require calibrated interpretation. Neither category showed a multiplicity-adjusted primary association with the composite outcome. The father-only group was especially small (*n* = 121), and its category-specific IV first stages were weak; those IV coefficients therefore receive no substantive interpretation. Larger parent-specific samples are needed to distinguish genuine heterogeneity from sampling imprecision.

The IV analyses provide limited but useful triangulation. Their first stages were strong for the binary and neither-parent contrasts, and second-stage estimates were positive across the two instrument constructions, but the raw-score estimates were imprecise and category-specific tests did not remain significant after Holm adjustment. Moreover, school-level parental noncoresidence may proxy local migration patterns, socioeconomic conditions, peer composition, school resources, or behavioral climate. The IV findings therefore support directional robustness under explicit assumptions rather than a stand-alone causal conclusion.

### 4.2. Parent–Child Interaction as Parallel Regulatory and Communicative Processes

The process findings clarify why physical presence and parenting practice should not be treated as interchangeable. Not living with both parents was associated more consistently with less frequent parent–child communication than with lower supervision. At the category level, communication was lower in the mother-only, father-only, and neither-parent groups, whereas the clearest reduction in supervision appeared for the neither-parent group. These patterns fit the different practical requirements of the two processes. Rules can sometimes be delegated to a resident parent or caregiver and sustained through established routines. Communication, by contrast, requires repeated exchanges with the parent whose experiences, advice, and emotional availability are being sought. Telephone and digital contact make those exchanges possible across distance, but they do not guarantee the same frequency, timing, or depth of discussion as everyday access (F. Wang et al., 2019; Xie et al., 2024).

Both interaction dimensions were inversely associated with deviant behavior in the joint outcome model. This finding is consistent with research relating behavioral control, parental knowledge, disclosure, and communication to adolescent conduct, but it also supports a more differentiated reading of that literature. Monitoring measures may combine parental rules, parental solicitation, adolescent disclosure, and parental knowledge, even though these components do not necessarily operate in the same way (Kapetanovic et al., 2019). Vaughan et al. (2022), for example, found a clear negative between-person association between parental monitoring and delinquent-peer affiliation but limited evidence that within-person changes in monitoring produced subsequent reductions in peer delinquency. Likewise, the longitudinal links among disclosure, parental knowledge, closeness, and delinquency were not uniformly reproduced in a South Korean sample (Rudy et al., 2023). These studies caution against treating “more monitoring” as a single universal mechanism. The present distinction between supervision and communication helps address that problem by separating parents’ regulation of behavioral domains from discussion of adolescents’ school and personal experiences.

The indirect-association results indicate that the two interaction processes are complementary statistical correlates. In the binary and neither-parent analyses, supervision and communication each contributed to the coefficient-product decomposition after multiplicity adjustment. These patterns are compatible with a structural–process account, but the concurrent measurement of coresidence, interaction, and behavior prevents them from establishing mediation or temporal mechanisms.

At the same time, communication should not be understood as the simple frequency of parental questioning. Its value depends on whether exchanges permit disclosure, convey responsiveness, and occur within a relationship that adolescents experience as supportive. Longitudinal evidence indicates that the family’s emotional climate conditions links between parent-driven and adolescent-driven communication and psychosocial functioning (Kapetanovic & Skoog, 2021). The present measure captures how often parents discuss school events, relationships, and private troubles, but not the tone, reciprocity, or perceived safety of those conversations. This measurement boundary helps explain why communication and supervision statistically account for only part of the coresidence association. Other processes—caregiver consistency, parent–caregiver coordination, peer affiliations, family conflict, economic strain, and adolescents’ own coping resources—may operate alongside them. Cross-sectional research has likewise identified social support and sleep quality as additional pathways linking left-behind experience with behavior problems (Ge et al., 2022), while recent rural longitudinal evidence points to distinct roles for psychological capital and perceived social support in the development of problem behavior (Huo et al., 2026).

The attenuation of the direct coresidence coefficient after both interaction measures entered the model is consequently best interpreted as partial statistical accounting, not as proof of complete mediation. A nonsignificant direct coefficient is not evidence that all other pathways have disappeared, and the shared cross-sectional measurement does not establish temporal ordering. What the complete a, b, c, c′, and ab structure does show is internally coherent: the coresidence arrangements most strongly associated with deviant behavior were also characterized by reduced parent–child interaction, and both interaction dimensions were related to the outcome in the expected direction.

### 4.3. Broader and Practical Implications

Although rural China provides the institutional setting, the findings address a broader family-development problem. Employment-related mobility, transnational migration, military deployment, rotational work, divorce, and other circumstances can separate adolescents from one or both parents. Across these settings, the key issue is not only whether a parent is geographically absent, but how families preserve parental availability through shared routines, communication, and coordination with resident caregivers. The concentration of the association in the neither-parent group suggests that the relevant vulnerability may arise when everyday guidance and relational access must both be maintained across distance, rather than from nontraditional family structure itself.

The practical implication is a dual-focus approach. Families separated by work or migration may benefit from predictable communication that goes beyond checking attendance or grades to include friendships, school experiences, and private concerns. At the same time, communication is unlikely to substitute fully for consistent behavioral expectations. Nonresident parents, resident caregivers, and adolescents need clear agreements about rules, responsibilities, and how emerging problems are shared. Schools can support this continuity by creating reliable channels through which caregivers and nonresident parents receive timely information and by ensuring that adolescents know which adult they can approach when family contact is difficult. Evidence that classroom and relational contexts are associated with aggression among left-behind adolescents further supports a family–school perspective (F. Yang et al., 2022). These evidence-informed targets provide concrete priorities for prospective family- and school-based intervention evaluation.

This perspective also avoids framing adolescents in mobile or nonresident-parent families as uniformly at risk. Most differences were modest, and the one-parent categories did not show the same adjusted pattern as the neither-parent category. The more precise conclusion is that family structure organizes opportunities and constraints, while family processes and wider relational resources shape how those conditions are experienced. Policies that facilitate family reunification may reduce structural separation, but when separation continues, strengthening both communicative and regulatory continuity provides an additional and potentially more immediately actionable direction.

### 4.4. Strengths, Limitations, and Future Directions

Several features strengthen the study. Primary analyses used one common sample and school-clustered inference; binary and four-category measures balanced parsimony with heterogeneous arrangements. The study includes raw-score, expanded-control, log-outcome, multiple-imputation, item-level, and IV sensitivity analyses, and uses Holm adjustment within the related testing families described above and in the Supplementary Materials. Communication was measured for both mothers and fathers and retained when reported for nonresident parents.

The findings should nevertheless be interpreted in light of several limitations. First, the analytic file is cross-sectional. The coefficient-product results identify statistical indirect associations, but they cannot determine whether reduced interaction preceded deviant behavior or whether adolescent behavior also changed parental supervision and communication. Methodological research shows that cross-sectional mediation estimates can differ from the longitudinal processes they are intended to represent (O’Laughlin et al., 2018). Multiwave designs should therefore model changes in coresidence, interaction, and behavior in temporal sequence and distinguish stable between-family differences from within-family change, as recent rural research has begun to do for other psychosocial resources (Huo et al., 2026).

Second, the focal measures were adolescent reports, making shared method variance, recall, and social-desirability bias possible. The deviant-behavior index also combines heterogeneous domains and showed modest internal consistency (α = 0.663). Item-level sensitivity analyses indicated that the binary association was most evident for profanity/verbal abuse and academic dishonesty after Holm adjustment, whereas other items were less precise. The composite is therefore best treated as a broad index of school-relevant norm violation, not a unitary clinical construct.

Third, current coresidence does not reveal why, when, or for how long a parent has been nonresident, nor does it identify the resident caregiver in the neither-parent group. Separation caused by labor migration may differ from separation following divorce, bereavement, illness, or other family transitions. Duration, age at first separation, visit schedules, remittances, caregiver identity, and parent–caregiver coordination are therefore important candidates for future analysis. Larger father-only samples would also permit more precise tests of parent-specific arrangements.

Fourth, complete-case analysis excluded 1231 of 4843 eligible adolescents, and retention varied across coresidence categories. Included and excluded participants differed on several observed characteristics. Multiple imputation preserved the principal binary and neither-parent patterns, but unobserved selection cannot be eliminated. Fifth, the school-level instruments had strong binary first stages, yet their exclusion restriction is not directly testable; the father-only instruments were weak. Finally, analyses were unweighted, so estimates describe the defined rural-hukou analytic sample rather than national prevalence.

## 5. Conclusions

Family coresidence was associated with rural adolescents’ reported deviant behavior, with the clearest multiplicity-adjusted category pattern among adolescents living with neither parent. The direction was supported by multiple-imputation and alternative-outcome analyses, while attenuation in expanded-control models and imprecision in IV analyses define the evidential boundary. Parental supervision and parent–child communication were distinct, complementary correlates that statistically accounted for part of the observed association without establishing temporal mediation.

The central implication is that family structure and family process should be examined jointly. Physical presence can facilitate parenting, yet its developmental significance depends partly on whether families preserve behavioral guidance and meaningful communication. For families in which parents and adolescents live apart, supporting coordinated supervision and regular, responsive conversation may help sustain the functions ordinarily made easier by coresidence. Longitudinal and multisource research is now needed to establish how changes in these processes unfold over time and to evaluate which family- and school-based supports are most effective across different forms of nonresident parenting.

## Figures and Tables

**Table 1:** Descriptive statistics for study variables (*N* = 3612).

Variable	Definition and Coding	Mean	SD	Min	Max
Deviant behavior	Sum of five items, 5–25	7.401	2.267	5.000	25.000
Not living with both parents	1 = yes, 0 = no	0.260	0.439	0.000	1.000
Mother-only coresidence	1 = yes, 0 = no; both-parent reference	0.090	0.287	0.000	1.000
Father-only coresidence	1 = yes, 0 = no; both-parent reference	0.033	0.180	0.000	1.000
Neither-parent coresidence	1 = yes, 0 = no; both-parent reference	0.136	0.343	0.000	1.000
Parental supervision	Sum of six items, 6–18	13.650	2.433	6.000	18.000
Parent–child communication	Sum of eight items, 8–24	16.378	4.018	8.000	24.000
Age	Years	13.671	0.794	11.000	18.000
Male	1 = male, 0 = female	0.505	0.500	0.000	1.000
Only child	1 = yes, 0 = no	0.263	0.440	0.000	1.000
Number of close friends (log)	ln(1 + number of close friends)	2.074	0.785	0.000	4.605
Academic achievement	Mean percentage across Chinese, mathematics, and English	63.751	19.010	1.667	96.000
Psychological distress	Mean of nine items, 1–5	2.195	0.795	1.000	5.000
Father’s educational attainment	1 = none to 7 = postgraduate or above	3.081	0.791	1.000	7.000
Mother’s educational attainment	1 = none to 7 = postgraduate or above	2.872	0.859	1.000	7.000
Good parental relationship	1 = yes, 0 = no	0.916	0.278	0.000	1.000
Father frequently drunk	1 = yes, 0 = no	0.086	0.280	0.000	1.000
Independent household toilet	1 = yes, 0 = no	0.821	0.383	0.000	1.000
Middle family economic condition	1 = yes, 0 = no; difficult reference	0.727	0.446	0.000	1.000
Affluent family economic condition	1 = yes, 0 = no; difficult reference	0.066	0.248	0.000	1.000
Low school-area misconduct	1 = yes, 0 = no; none reference	0.807	0.394	0.000	1.000
High school-area misconduct	1 = yes, 0 = no; none reference	0.111	0.314	0.000	1.000
Central China	1 = yes, 0 = no; eastern reference	0.270	0.444	0.000	1.000
Western China	1 = yes, 0 = no; eastern reference	0.217	0.412	0.000	1.000

**Table 2:** Family coresidence and untransformed deviant-behavior scores among rural adolescents.

Variable	(1) Binary: Core	(2) Binary: Expanded	(3) Four-Category: Core	(4) Four-Category: Expanded
Not living with both parents	0.312 ** (0.100)	0.155 (0.081)		
Mother only			0.091 (0.108)	−0.018 (0.096)
Father only			0.597 (0.280)	0.369 (0.266)
Neither parent			0.388 ** (0.121)	0.219 (0.102)
Core covariates	Yes	Yes	Yes	Yes
Expanded controls	No	Yes	No	Yes
Observations	3612	3612	3612	3612
R^2^	0.060	0.174	0.062	0.175

Notes: Coefficients are unstandardized OLS estimates; school-clustered standard errors are in parentheses. Core covariates are sex, age, only-child status, parental education, and region. Expanded models add the remaining covariates described in Section 2.2.4. Stars in binary models use two-sided *p* values; stars in four-category models use Holm-adjusted *p* values across the three contrasts. ** *p* < 0.01.

**Table 3:** Instrumental-variable sensitivity analyses for binary family coresidence and raw deviant behavior.

Variable	(1) First Stage Coresidence	(2) Second Stage Deviant Behavior	(3) First Stage Coresidence	(4) Second Stage Deviant Behavior
Not living with both parents		0.644 (0.447)		0.691 (0.460)
Same-school peer share not living with both parents	1.087 *** (0.043)			
Same-school share excluding own class			1.054 *** (0.046)	
Core covariates	Yes	Yes	Yes	Yes
First-stage partial R^2^	0.118		0.113	
Cluster-robust first-stage χ^2^	648.22 ***		524.86 ***	
Observations	3612	3612	3607	3607
Outcome-model R^2^		0.056		0.054
Instrument construction	Leave-one-out school share	Leave-one-out school share	Outside-class school share	Outside-class school share

Notes: Columns 1–2 use the leave-one-out same-school share; Columns 3–4 use the outside-class school share. All models include core covariates. Second-stage coefficients are treated as sensitivity estimates. School-clustered standard errors are in parentheses. *** *p* < 0.001.

**Table 4:** Category-specific IV diagnostics for family coresidence and raw deviant behavior.

Variable	(1) First Stage Focal Category	(2) Second Stage Deviant Behavior	(3) First Stage Focal Category	(4) Second Stage Deviant Behavior
**Panel A: Mother only versus both parents**				
Mother only		0.922 (1.202)		0.920 (1.295)
Same-school focal-category share	1.348 *** (0.116)			
Focal-category share excluding own class			1.202 *** (0.135)	
Core covariates	Yes	Yes	Yes	Yes
First-stage partial R^2^	0.038		0.033	
Cluster-robust first-stage χ^2^	134.81 ***		79.65 ***	
Observations	3000	3000	2995	2995
Outcome-model R^2^		0.045		0.044
**Panel B: Father only versus both parents**				
Father only		14.741 (10.260)		34.590 (34.665)
Same-school focal-category share	0.575 * (0.254)			
Focal-category share excluding own class			0.261 (0.226)	
Core covariates	Yes	Yes	Yes	Yes
First-stage partial R^2^	0.003		0.001	
Cluster-robust first-stage χ^2^	5.13 *		1.34	
Observations	2795	2795	2790	2790
Outcome-model R^2^		−1.554		−9.275
**Panel C: Neither parent versus both parents**				
Neither parent		0.853 (0.379)		0.852 (0.372)
Same-school focal-category share	1.324 *** (0.044)			
Focal-category share excluding own class			1.311 *** (0.052)	
Core covariates	Yes	Yes	Yes	Yes
First-stage partial R^2^	0.162		0.157	
Cluster-robust first-stage χ^2^	923.81 ***		638.70 ***	
Observations	3165	3165	3160	3160
Outcome-model R^2^		0.054		0.053
Instrument version	Same-school	Same-school	Outside-class	Outside-class

Notes: Each panel compares one focal category with both-parent coresidence. Core covariates are included. Second-stage stars use Holm-adjusted *p* values across the six category-specific tests; first-stage stars use two-sided unadjusted *p* values. The father-only first stages are weak and those second-stage estimates should not be interpreted substantively. * *p* < 0.05, *** *p* < 0.001.

**Table 5:** Exploratory parallel indirect associations of family coresidence, parent–child interaction, and raw deviant behavior.

Coresidence Contrast and Pathway	(1) a Path	(2) b Path	(3) Indirect ab	(4) 95% Bootstrap CI
**Panel A: Binary family coresidence**				
Not living with both parents: Parental supervision	−0.122 ** (0.042)	−0.381 *** (0.055)	0.046 *	[0.013, 0.079]
Not living with both parents: Parent–child communication	−0.246 *** (0.045)	−0.290 *** (0.051)	0.071 *	[0.038, 0.110]
Not living with both parents: Combined indirect association			0.118 *	[0.066, 0.172]
**Panel B: Four-category family coresidence (reference: both parents)**				
Common b path: Parental supervision		−0.380 *** (0.055)		
Common b path: Parent–child communication		−0.288 *** (0.051)		
Mother only: Parental supervision	−0.065 (0.057)		0.025	[−0.018, 0.068]
Mother only: Parent–child communication	−0.174 ** (0.057)		0.050 *	[0.014, 0.091]
Mother only: Combined indirect association			0.075	[0.006, 0.148]
Father only: Parental supervision	−0.222 * (0.102)		0.084	[0.008, 0.166]
Father only: Parent–child communication	−0.407 *** (0.097)		0.117 *	[0.055, 0.195]
Father only: Combined indirect association			0.201 *	[0.087, 0.318]
Neither parent: Parental supervision	−0.134 ** (0.049)		0.051 *	[0.012, 0.094]
Neither parent: Parent–child communication	−0.253 *** (0.057)		0.073 *	[0.036, 0.121]
Neither parent: Combined indirect association			0.124 *	[0.065, 0.194]

Notes: a paths predict standardized supervision or communication; b paths are their coefficients in the joint raw-outcome equation. Indirect-association confidence intervals use 1999 school-cluster bootstrap replications. Stars for a and b paths use two-sided *p* values; stars for indirect associations use Holm-adjusted *p* values across 12 tests. * *p* < 0.05, ** *p* < 0.01, *** *p* < 0.001.

**Table 6:** Total and direct associations of family coresidence with raw deviant behavior.

Coresidence Contrast	(1) Total Association c	(2) Direct Association c′	(3) Change c − c′	(4) Observations
**Panel A: Binary family coresidence**				
Not living with both parents	0.312 ** (0.100)	0.194 * (0.093)	0.118	3612
**Panel B: Four-category family coresidence (reference: both parents)**				
Mother only	0.091 (0.108)	0.016 (0.103)	0.075	3612
Father only	0.597 (0.280)	0.396 (0.267)	0.201	3612
Neither parent	0.388 ** (0.121)	0.264 (0.115)	0.124	3612

Notes: Models include core covariates. The total association (c) excludes supervision and communication; the direct association (c′) includes both standardized interaction measures. Changes equal the combined product terms in Table 5. School-clustered standard errors are in parentheses. Binary-model stars use two-sided *p* values; category-model stars use Holm-adjusted *p* values across the three contrasts. * *p* < 0.05, ** *p* < 0.01.

## Data Availability

The third-party data analyzed in this study were obtained from the China Education Panel Survey, administered by the National Survey Research Center at Renmin University of China. The data are available for academic research through the China National Social Survey Data Archive (https://www.cnsda.org/index.php, accessed on 20 September 2026) subject to registration and the data provider’s terms. In accordance with those terms, the authors do not redistribute the raw microdata. Analysis code and machine-readable model-output files are provided in Supplementary File S2.

## Supplementary Materials

The following supporting information can be downloaded at https://www.mdpi.com/article/10.3390/bs16101767/s1, Supplementary File S1 contains the following tables: Table S1: Analytic sample flow; Table S2: Variable-specific missingness among eligible rural-hukou adolescents (*N* = 4843); Table S3: Complete-case retention by family-coresidence category; Table S4: Item diagnostics for the deviant-behavior index (*N* = 3612); Table S5: Log-outcome sensitivity models; Table S6: Multiple-imputation sensitivity analysis (20 imputations; *N* = 4782); Table S7: Item-level sensitivity analyses with Holm adjustment; Table S8: Expanded-control binary IV sensitivity analyses; Table S9: Included-versus-excluded comparisons on observed characteristics. Supplementary File S2 contains analysis code, machine-readable output tables, an environment specification, and a reproducibility readme. CEPS raw microdata are not redistributed.

## References

[B1-behavsci-16-01767] Akezhuoli H., Lu J., Zhao G., Xu J., Wang M., Wang F., Li L., Zhou X. (2022). Mother’s and father’s migrating in China: Differing relations to mental health and risk behaviors among left-behind children. Frontiers in Public Health.

[B2-behavsci-16-01767] Chen X., Li L., Lv G., Li H. (2021). Parental behavioral control and bullying and victimization of rural adolescents in China: The roles of deviant peer affiliation and gender. International Journal of Environmental Research and Public Health.

[B3-behavsci-16-01767] Fellmeth G., Rose-Clarke K., Zhao C., Busert L. K., Zheng Y., Massazza A., Sonmez H., Eder B., Blewitt A., Lertgrai W., Orcutt M., Ricci K., Mohamed-Ahmed O., Burns R., Knipe D., Hargreaves S., Hesketh T., Opondo C., Devakumar D. (2018). Health impacts of parental migration on left-behind children and adolescents: A systematic review and meta-analysis. The Lancet.

[B4-behavsci-16-01767] Ge M., Yang M., Sheng X., Zhang L., Zhang K., Zhou R., Ye M., Cao P., Sun Y., Zhou X. (2022). Left-behind experience and behavior problems among adolescents: Multiple mediating effects of social support and sleep quality. Psychology Research and Behavior Management.

[B5-behavsci-16-01767] Huang Y., McKeown S. (2022). Examining the association between family environment and adolescent delinquent behaviours in China. Journal of Child and Family Studies.

[B6-behavsci-16-01767] Huo Z., Tan T., Yang N., Wu J. (2026). The impact of psychological capital and perceived social support on the development of problem behaviors among rural adolescents: A cross-lagged study. Behavioral Sciences.

[B7-behavsci-16-01767] Kapetanovic S., Boele S., Skoog T. (2019). Parent–adolescent communication and adolescent delinquency: Unraveling within-family processes from between-family differences. Journal of Youth and Adolescence.

[B8-behavsci-16-01767] Kapetanovic S., Skoog T. (2021). The role of the family’s emotional climate in the links between parent–adolescent communication and adolescent psychosocial functioning. Research on Child and Adolescent Psychopathology.

[B9-behavsci-16-01767] Ksinan A. J., Vazsonyi A. T. (2016). Longitudinal associations between parental monitoring discrepancy and delinquency: An application of the latent congruency model. Journal of Youth and Adolescence.

[B10-behavsci-16-01767] Lu Y., Zhang R., Du H. (2021). Family structure, family instability, and child psychological well-being in the context of migration: Evidence from sequence analysis in China. Child Development.

[B11-behavsci-16-01767] Ma Y., Li Y., Zhang Y., Xie X., Lin X., Fu H., Huang M., Zhou W., Ji J. S., Chen R., Liu S., Yu Y., Tang J. (2024). Labor migration of parents and aggression among their offspring in China. JAMA Network Open.

[B12-behavsci-16-01767] O’Laughlin K. D., Martin M. J., Ferrer E. (2018). Cross-sectional analysis of longitudinal mediation processes. Multivariate Behavioral Research.

[B13-behavsci-16-01767] Pinquart M. (2017). Associations of parenting dimensions and styles with externalizing problems of children and adolescents: An updated meta-analysis. Developmental Psychology.

[B14-behavsci-16-01767] Rudy D., Han S., Yu M. (2023). South Korean adolescents’ delinquency, disclosure, parental knowledge, parent–child closeness, and delinquent peer associations. Journal of Research on Adolescence.

[B15-behavsci-16-01767] Vaughan E. P., Speck J. S., Frick P. J., Robertson E. L., Ray J. V., Thornton L. C., Wall Myers T. D., Steinberg L., Cauffman E. (2022). Longitudinal associations of parental monitoring and delinquent peer affiliation: The potential influence of parental solicitation and monitoring rules. Journal of Adolescence.

[B16-behavsci-16-01767] Wang F., Lin L., Xu M., Li L., Lu J., Zhou X. (2019). Mental health among left-behind children in rural China in relation to parent–child communication. International Journal of Environmental Research and Public Health.

[B17-behavsci-16-01767] Wang N., Liu Y., Dang R., Liu S., Shi Y. (2026). The impact of parental absence on adolescents’ health risk behaviors: Evidence from the China Education Panel Survey. Emerging Markets Finance and Trade.

[B18-behavsci-16-01767] Xie Q.-W., Chen R., Wang K., Lu J., Wang F., Zhou X. (2024). Associations of latent patterns of parent–child communication with communication quality and mental health outcomes among Chinese left-behind children. BMC Public Health.

[B19-behavsci-16-01767] Xu T., Ren J. (2025). Parent–child relationships, parental control, and adolescent mental health: An empirical study based on CEPS 2013–2014 survey data. Behavioral Sciences.

[B20-behavsci-16-01767] Yang F., Jin Z., He J., Han B., Huang X., Chen K., Wang J. (2022). Aggressive behaviors and associated factors in Chinese left-behind adolescents: A cross-sectional study. BMC Pediatrics.

[B21-behavsci-16-01767] Yang Y., Jiang J. (2023). Influence of family structure on adolescent deviant behavior and depression: The mediation roles of parental monitoring and school connectedness. Public Health.

[B22-behavsci-16-01767] Zapf H., Boettcher J., Haukeland Y., Orm S., Coslar S., Fjermestad K. (2024). A systematic review of the association between parent–child communication and adolescent mental health. JCPP Advances.

