# Supplementary material for: Family Coresidence, Parent–Child Interaction, and Deviant Behavior Among Rural Chinese Adolescents
Source: Behav Sci (Basel). 2026 Sep 28;16(10):1767. doi: 10.3390/bs16101767 (PMC13701682; doi:10.3390/bs16101767)
Supplement: Supplementary file 1 [file behavsci-16-01767-s001.zip › Supplementary_File_S1_Additional_Analyses.pdf]

Supplementary File S1

Family Coresidence, Parent–Child Interaction, and Deviant Behavior among Rural Chinese Adolescents | Manuscript ID: behavsci-4587502

This supplement reports analyses added in response to peer review. All regression standard errors are clustered by school unless stated otherwise. Two-sided α = 0.05 is used.

Table S1. Analytic sample flow

Stage	N	Excluded from previous stage
Original cohort records	9449	—
Eligible agricultural-hukou adolescents	4843	4606.0
Observed coresidence and five-item outcome	4782	61.0
Common complete-case analytic sample	3612	1170.0

The difference between 4,843 eligible cases and 3,612 complete cases is 1,231; 61 lacked the five-item outcome and 1,170 additional cases were incomplete on another common-sample variable.

Table S2. Variable-specific missingness among eligible rural-hukou adolescents (N = 4,843)

Variable	Missing n	Missing %	Observed n
Deviant-behavior total score	61	1.26	4782
Not living with both parents	0	0.00	4843
Four-category family coresidence	0	0.00	4843
Parental supervision	64	1.32	4779
Parent–child communication	356	7.35	4487
Male	0	0.00	4843
Age	134	2.77	4709
Only child	78	1.61	4765
Number of close friends (log)	227	4.69	4616
Academic achievement	77	1.59	4766
Psychological distress	136	2.81	4707
Father's education	208	4.29	4635
Mother's education	223	4.60	4620
Good parental relationship	55	1.14	4788
Father frequently drunk	22	0.45	4821
Independent household toilet	193	3.99	4650
Middle family economic condition	15	0.31	4828
Affluent family economic condition	15	0.31	4828
Low school-area misconduct	0	0.00	4843
High school-area misconduct	0	0.00	4843
Central China	0	0.00	4843
Western China	0	0.00	4843

Table S3. Complete-case retention by family-coresidence category

Category	Eligible n	Complete-case n	Excluded n	Retained %
Both parents	3483	2674	809	76.77
Mother only	472	326	146	69.07

Category	Eligible n	Complete-case n	Excluded n	Retained %
Father only	193	121	72	62.69
Neither parent	695	491	204	70.65

Table S4. Item diagnostics for the deviant-behavior index (N = 3,612)

Item	Mean	SD	Corrected item–total r	α if deleted
Profanity or verbal abuse	2.276	0.965	0.434	0.629
Fighting	1.342	0.676	0.481	0.582
Bullying weaker classmates	1.141	0.472	0.445	0.616
Skipping class or school	1.084	0.400	0.360	0.647
Copying homework or examination cheating	1.558	0.801	0.492	0.574

Overall Cronbach's α = 0.663. The index is interpreted as a broad behavioral domain rather than a unidimensional clinical scale.

Table S5. Log-outcome sensitivity models

Model	Contrast	b	SE	95% CI	p	Holm p
log_binary_core	nonboth	0.039	0.011	[0.017, 0.062]	0.001	
log_binary_full	nonboth	0.020	0.009	[0.001, 0.038]	0.034	
log_four_core	mother_only	0.016	0.013	[-0.010, 0.042]	0.235	0.235
log_four_core	father_only	0.067	0.028	[0.010, 0.123]	0.021	0.042
log_four_core	neither_parent	0.048	0.014	[0.020, 0.076]	0.001	0.003
log_four_full	mother_only	0.001	0.012	[-0.022, 0.024]	0.917	0.917
log_four_full	father_only	0.041	0.027	[-0.014, 0.095]	0.140	0.281
log_four_full	neither_parent	0.027	0.012	[0.003, 0.050]	0.025	0.076

Logged-score coefficients are not interpreted as percentage differences because the underlying 5–25 score has no meaningful ratio zero.

Table S6. Multiple-imputation sensitivity analysis (20 imputations; N = 4,782)

Model	Contrast	b	SE	95% CI	p	Holm p
binary	nonboth	0.243	0.073	[0.100, 0.387]	0.001	
four_category	mother_only	0.028	0.111	[-0.190, 0.246]	0.799	0.799
four_category	father_only	0.365	0.200	[-0.027, 0.758]	0.068	0.137
four_category	neither_parent	0.359	0.094	[0.175, 0.543]	<0.001	<0.001

The imputation sample includes adolescents with observed family coresidence and complete five-item outcome data. Estimates are pooled using Rubin's rules.

Table S7. Item-level sensitivity analyses with Holm adjustment

Behavior item	Specification	Contrast	b	SE	p	Holm p
Profanity or verbal abuse	binary	nonboth	0.102	0.038	0.009	0.034
Profanity or verbal abuse	four_category	mother_only	0.079	0.049	0.111	1.000
Profanity or verbal abuse	four_category	father_only	0.130	0.085	0.132	1.000
Profanity or verbal abuse	four_category	neither_parent	0.111	0.051	0.032	0.450
Fighting	binary	nonboth	0.047	0.030	0.127	0.382
Fighting	four_category	mother_only	0.027	0.043	0.533	1.000
Fighting	four_category	father_only	0.120	0.072	0.097	1.000
Fighting	four_category	neither_parent	0.041	0.038	0.275	1.000
Bullying weaker classmates	binary	nonboth	0.014	0.019	0.459	0.918
Bullying weaker classmates	four_category	mother_only	-0.022	0.025	0.387	1.000

Behavior item	Specification	Contrast	b	SE	p	Holm p
Bullying weaker classmates	four_category	father_only	0.106	0.058	0.069	0.891
Bullying weaker classmates	four_category	neither_parent	0.015	0.026	0.553	1.000
Skipping class or school	binary	nonboth	0.012	0.020	0.563	0.918
Skipping class or school	four_category	mother_only	-0.020	0.024	0.414	1.000
Skipping class or school	four_category	father_only	0.078	0.060	0.198	1.000
Skipping class or school	four_category	neither_parent	0.016	0.024	0.509	1.000
Copying homework or examination cheating	binary	nonboth	0.137	0.035	<0.001	0.001
Copying homework or examination cheating	four_category	mother_only	0.027	0.049	0.582	1.000
Copying homework or examination cheating	four_category	father_only	0.163	0.091	0.076	0.915
Copying homework or examination cheating	four_category	neither_parent	0.204	0.039	<0.001	<0.001

Holm adjustment is applied separately to the five binary-exposure tests and the 15 four-category tests. The binary association survives adjustment for profanity/verbal abuse and copying homework/examination cheating.

Table S8. Expanded-control binary IV sensitivity analyses

Instrument	N	Second-stage b	SE	95% CI	p	First-stage χ^2	Partial R^2
full_same_school_loo	3612	0.621	0.375	[-0.113, 1.356]	0.097	683.38	0.111
full_outside_class	3607	0.638	0.379	[-0.104, 1.381]	0.092	525.25	0.106

These estimates are sensitivity/triangulation results and do not resolve the exclusion-restriction concern.

Table S9. Included-versus-excluded comparisons on observed characteristics

Variable	Included mean	Excluded mean	SMD	Welch p
Deviant-behavior total score	7.401	7.845	-0.171	<0.001
Not living with both parents	0.260	0.343	-0.182	<0.001
Mother Only	0.090	0.119	-0.093	0.006
Father Only	0.033	0.058	-0.119	0.001
Neither Parent	0.136	0.166	-0.083	0.013
Male	0.505	0.616	-0.225	<0.001
Age	13.671	13.843	-0.206	<0.001
Only child	0.263	0.271	-0.017	0.613
Number of close friends (log)	2.074	2.183	-0.133	<0.001
Academic achievement	63.751	55.305	0.424	<0.001
Psychological distress	2.195	2.279	-0.100	0.005
Father's education	3.081	3.030	0.061	0.095
Mother's education	2.872	2.835	0.040	0.272
Good parental relationship	0.916	0.879	0.121	0.001
Father frequently drunk	0.086	0.105	-0.065	0.054
Independent household toilet	0.821	0.776	0.112	0.002
Middle family economic condition	0.727	0.677	0.110	0.001
Affluent family economic condition	0.066	0.054	0.048	0.141
Low school-area misconduct	0.807	0.758	0.120	<0.001
High school-area misconduct	0.111	0.119	-0.026	0.429
Central China	0.270	0.343	-0.157	<0.001
Western China	0.217	0.252	-0.081	0.015

Observed n varies in the excluded group because missingness is variable-specific. SMD = standardized mean difference (included minus excluded).
